# Dermal fibroblasts are the key sensors of aseptic skin inflammation through interleukin 1 release by lesioned keratinocytes

**DOI:** 10.3389/fimmu.2022.984045

**Published:** 2022-10-03

**Authors:** Sevda Cordier-Dirikoc, Nathalie Pedretti, Julien Garnier, Sandrine Clarhaut-Charreau, Bernhard Ryffel, Franck Morel, François-Xavier Bernard, Valérie Hamon de Almeida, Jean-Claude Lecron, Jean-François Jégou

**Affiliations:** ^1^ Qima-Bioalternatives (Qima Life Sciences), Gençay, France; ^2^ Université de Poitiers, Laboratoire Inflammation, Tissus Epithéliaux et Cytokines (LITEC), UR15560, Poitiers, France; ^3^ INEM, CNRS, UMR 7355, Orléans, France; ^4^ Service d’Immunologie et Inflammation, CHU de Poitiers, Poitiers, France

**Keywords:** keratinocytes, fibroblasts, IL-1, skin, sterile inflammation, epidermal lesion

## Abstract

IL-1 plays a crucial role in triggering sterile inflammation following tissue injury. Although most studies associate IL-1 release by injured cells to the recruitment of neutrophils for tissue repair, the inflammatory cascade involves several molecular and cellular actors whose role remains to be specified. In the present study, we identified dermal fibroblasts among the IL-1R1-expressing skin cells as key sensors of IL-1 released by injured keratinocytes. After *in vitro* stimulation by recombinant cytokines or protein extracts of lysed keratinocytes containing high concentrations of IL-1, we show that dermal fibroblasts are by far the most IL-1-responsive cells compared to keratinocytes, melanocytes and endothelial cells. Fibroblasts have the property to respond to very low concentrations of IL-1 (from 10 fg/ml), even in the presence of 100-fold higher concentrations of IL-1RA, by increasing their expression of chemokines such as IL-8 for neutrophil recruitment. The capacity of IL-1-stimulated fibroblasts to attract neutrophils has been demonstrated both *in vitro* using cell migration assay and *in vivo* using a model of superficial epidermal lesion in IL-1R1-deficient mice which harbored reduced expression of inflammatory mediators and neutrophil skin infiltration. Together, our results shed a light on dermal fibroblasts as key relay cells in the chain of sterile inflammation induced after epidermal lesion.

## Introduction

Skin is the outermost tissue of the human body that represents a primary interface with the external environment. It provides a protective barrier against mechanical, thermal and physical injuries and constitutes the first line of defense against pathogens. In this multilayered tissue, the epidermis plays a crucial role in the natural barrier function, in association with a wide repertoire of immune cells both from the innate and adaptive systems which contribute to host defense and the maintenance of skin homeostasis (1).

After a primary epidermal lesion in aseptic condition, keratinocytes have the capacity to trigger a sequence of events involving a wide diversity of molecular and cellular actors to initiate a dedicated inflammatory response, leading to local protection and repair mechanisms. The role of keratinocytes has been widely studied in the pathogenesis of chronic skin inflammatory pathologies such as psoriasis or atopic dermatitis (2, 3). However, much less is known about the contribution of keratinocytes in the early events of the innate immune response after a primary epidermal lesion and the downstream mechanisms that contribute to the development of a sterile inflammation characterized by the infiltration of injured tissues by immune cells. In a pathogen-free context, this epidermal innate immune response can be induced after a cellular stress by the passive release of alarmins such as heat-shock proteins (HSPs), adenosine triphosphate (ATP), S100 proteins or IL-33, which activate sterile inflammation (4). Among these mediators, interleukin 1α (IL-1α) is also considered as an alarmin and it has been known for a long time that keratinocytes are a potent source of proIL-1α, as well as proIL-1β (5, 6), which are both matured and released under their active forms in response to different cellular stresses to trigger sterile inflammation. IL-1α and IL-1β are both leader cytokines of the IL-1 family which encompasses 11 members, including 7 agonists with proinflammatory activities (IL-1α, IL-1β, IL-18, IL-33, IL-36α, IL-36β, andIL-36γ) and 4 antagonists (IL-1RA, IL-18BP, IL-37, and IL-38) with anti-inflammatory activities (7, 8). Keratinocytes were also reported to express and secrete IL-18 (9), IL-33 (10) and IL-36α/β/γ (11) in inflammatory conditions, but most studies reported a predominant contribution of IL-1α and IL-1β to cutaneous sterile inflammation. Thus, both cytokines were described be upregulated and secreted by damaged keratinocytes after UVB exposure (12, 13). IL-1α represents a signal for genotoxic stress when keratinocytes and fibroblasts are exposed to various DNA damage agents, thus initiating a sterile inflammatory cascade that contributes to efficient tissue repair and wound healing (14). The role of IL-1α and IL-1β has also been investigated in hypoxia-induced sterile inflammation, both *in vitro* and *in vivo* (15). In this study, Rider *et al.* demonstrated the contribution of the proIL-1α and mature IL-1β released by necrotic keratinocytes to recruit sequentially neutrophils and macrophages, respectively. In another mouse model of intraperitoneal sterile inflammation after injured cells injection, the acute neutrophilic inflammatory response required IL-1α and the IL-1R1-Myd88 pathway (16).

Although most studies converge to a central role of IL-1 released by injured cells to trigger sterile inflammation and the recruitment of neutrophils, less is known about the potential target cells in the skin that express IL-1 receptors and that could act as intermediate cells contributing to attract neutrophils at the site of tissue injury. In the present study, we have investigated the role of IL-1 in the induction of aseptic cutaneous inflammation after primary epidermal lesion by integrating the different skin compartments. We have analyzed and compared the expression of cytokines of the IL-1 family and their receptors in a panel of skin and immune cells, allowing us to confirm that keratinocytes are the main source of IL-1α/β and to identify dermal fibroblasts as central relay cells, highly sensitive to low levels of IL-1 α/β, even in the presence of high concentrations of the antagonist IL-1RA, and able to promote skin inflammation through the release of neutrophil-attracting chemokines.

## Materials and methods

### Cell cultures, cytokines and reagents

Primary normal human epidermal keratinocytes (NHEK), normal human dermal fibroblasts (NHDF), normal human epidermal melanocytes (NHEM) and human dermal microvascular endothelial cells (HMVEC) were isolated from human skin and cultured as described in supplementary materials and methods (17–19). Peripheral blood mononuclear cells (PBMC) and polymorphonuclear neutrophils (PMN) were typically isolated from whole blood of healthy adult volunteers using Ficoll density gradient centrifugation. Subsequently, CD4^+^ T cells and CD14^+^ monocytes were isolated from PBMC through positive selection by magnetic cell sorting using MACS isolation kits (Miltenyi Biotec). Reconstituted human skins (RHS) were obtained from Qima-Bioalternatives (Gençay, France). Briefly, a collagen (from rat tail, Institut de Biotechnologies Jacques Boy) lattice containing fibroblasts is cast in a 0.5 cm² culture insert (Millipore). After polymerization, keratinocytes are seeded onto the lattice and the latter is incubated at 37°C/5% CO_2_ for 48 h. The medium in the inserts is then removed allowing the reconstructed skins to be placed in an air/liquid interface condition. The reconstructed skins are then grown for 7 days, changing the medium under the inserts every 2 or 3 days. The epidermal surface of RHS was then scarified with a scalpel under sterile condition and supernatants were collected 48 h after scarification for IL-8 measurements.

All recombinant cytokines (IL-1α, IL-1β, IL-36γ, IL-18, IL-33) were purchased from R&D systems. Anti-IL-1R1 polyclonal antibody (PA5-46930) and goat IgG isotype control were purchased from Thermo Fischer scientific.

ELISA for the detection of human IL-1α, IL-1β, IL-6, IL-8, IL-36γ, IL-18, IL-33, IL-1Ra, IL-18BPa, IL-36Ra and mouse CXCL-1, in cell culture supernatant or keratinocyte extract was carried out according to the manufacturer’s instructions (R&D Systems).

### RNA isolation and transcriptional analysis

Total RNA was extracted from cell cultures or mouse skin tissues using TriPure Isolation Reagent^®^ (Roche, Merck) and treated with DNase I (0.05 U/ml; Clontech). RNA was reverse-transcribed with « Transcriptor Reverse Transcriptase » (Roche) and transcripts were amplified and quantified using the LightCycler-FastStart DNA Master Plus SYBR Green I kit on a LightCycler 480 instrument (Roche). Oligonucleotides were designed with Primer 3 and-Blast software and purchased from Merck (Sigma-Aldrich) or Eurogentec. Primer sequences to amplify human IL-8 were 5’-CTCTTGGCAGCCTTCCTG-3’(Forward) and 5’-TTGGGGTCCAGACAGAGC-3’(Reverse). Forward and reverse primer sequences for mouse gene amplification were indicated in **Supplemental Table 1**. Samples were normalized to the expression of the control housekeeping gene GAPDH.

For Affymetrix analysis, RNA was extracted using the NucleoSpin RNA kit (Macherey-Nagel), according to the manufacturer’s instructions. The concentration and the quality of extracted RNA was determined for each sample using a 2100 bioanalyzer system (Agilent technologies). Probes were prepared and hybridized to the Affymetrix Human Genome U219 Array as recommended by the manufacturer. Equal amount and quality of RNA was used for each sample, constituting the first step of normalization. Data were obtained from measurements of the relative signal strength for probes containing ~30,000 transcripts. The raw intensity values are background corrected, log2 transformed and then quantile normalized according to the RMA algorithm. Then, a linear model is fit to the normalized data to obtain an expression measure for each probe set on each array. Data were submitted to the Gene Expression Omnibus Database (GSE111191) and to the Database for Annotation, Visualization and Integrated Discovery (DAVID) for clustering analysis (20).

### Keratinocyte extract preparation

NHEK were seeded at 80,000 cells/cm2 in T25 Flasks in supplemented keratinocyte-SFM medium. The culture medium was renewed 24 hours later. The next day, the medium was replaced by keratinocyte-SFM medium containing no supplements and cells were incubated for 48 hours (8 ml/T25 Flask). The cell-free culture supernatant was collected and stored at -80 C until use. Cells were trypsinized, washed in PBS solution and then centrifuged. The cell pellet was lysed using freeze/thaw procedure and sonication in 8 ml of ultrapure water for each flask (this volume corresponding to the same volume as the initial culture medium volume). The obtained cell lysate was aliquoted and stored at -80 C until use. The concentration of IL-1α was determined for each keratinocyte extract and normalized from one preparation to another.

### Cell stimulation with cytokines or keratinocyte extract

NHEK, NHDF, NHEM and HMVEC were all seeded at 150,000 cells/well in 24-well plates (Nunc). After 24 hours of incubation, the culture medium was renewed or replaced by assay medium, and cells were incubated for 24 hours. The next day, cells were incubated for 48 hours in presence or not of the cytokines or keratinocyte extract. On NHDF, keratinocyte extract was also tested in presence of an anti-IL1R1 neutralizing polyclonal antibody or the isotype control.

Blood cells were seeded in all assays at 200,000 cells/well in 96-well plates (1x10^6^ cells/ml) and incubated for 48 hours in presence or not of the stimulants (interleukins of IL1Fm or keratinocyte extract). The treatments with the stimulants were performed one hour after the seeding for 48 h.

At the end of stimulation, cell culture supernatants were harvested for ELISA quantification of IL-8 and/or IL-6 and when necessary and cell layers were used for RNA extraction and gene expression profiling.

### Cell migration assay

PMN migration was assessed using Boyden chamber transmigration system. Briefly, PMN freshly isolated from whole blood, were labeled using calcein-AM (Invitrogen, Thermo Fischer scientific), seeded onto the upper compartment of 3 µm-porous membrane inserts. Culture medium containing chemoattractant reference IL-8 or fibroblast culture supernatants was placed in the lower compartment and the cells were incubated for 90 minutes. Cells which have migrated through the membrane were quantified by measuring the fluorescence intensity (λ_exc_ 485 nm, λ_em_ 530 nm) of the recipient compartment using a fluorescent microplate reader (Spectramax, Molecular Devices).

### *In vivo* experiments

Male and female wild-type and IL-1R1-deficient C57Bl/6 mice were bred in the specific pathogen-free animal facility at CNRS (TAAM UPS44, Orleans, France). Mice were provided food and water ad libitum and maintained on a 12 h light/dark cycle. Experimental procedures were approved by French Government’s ethical and animal experiment regulations and were submitted to the ethics committee for animal Experimentation of “CNRS Campus Orleans” (CCO) under number APAFIS#30196-2019021818223038.

For experiments, 8–14-week-old mice were transferred to experimental animal facility. Under anesthesia by intraperitoneal injection of a xylazine (10 mg/mL)/ketamine (1 mg/mL) solution (100 μL/10 g of animal weight), the back skin of mice was shaved, and remaining hairs were removed using a depilatory cream (Veet@, Reckitt Benckiser). The skin was antiseptically cleaned before gentle scarification with a sterile scalpel for epidermal lesion (star-shaped scar to facilitate the observation of lesions on skin sections for subsequent immunohistological analysis). Twenty-four hours later, mice were euthanized and both lesioned back skin and non-lesioned skin were collected and snap-frozen in liquid nitrogen and stored at -80°C for further transcriptional and immunohistofluorescence analysis.

### Immunohistofluorescence

Immunohistofluorescence experiments were performed on cryopreserved mouse skin samples. Five-micrometer sections were cut from a tissue block. Sections were fixed in acetone/methanol and staining was performed using monoclonal antibodies against Mouse Gr1 (1:100; BD Biosciences; 550291) and vimentin (1:100; Abcam; ab92547). These stainings were detected using appropriate secondary antibodies (goat anti-rat IgG Alexa 488 conjugated secondary antibody for Gr1 and Alexa 568-conjugated goat anti-rabbit for vimentin). Sections were observed using a NIKON Ci microscope. The images were captured using a NIKON DS-Ri2 and processed with NIS Elements software.

For quantitative analyses, the intensity of Gr1 staining was measured in five representative areas for each section using the Image J software (National Institute of Health, USA).

### Statistical analyses

Statistical analysis of significance was calculated using either Mann & Whitney’s test or for *in vivo* experiments, the Kruskal-Wallis one-way ANOVA test with a Bonferroni post-test. The p values < 0.05 were considered as significant, and all data are represented as mean ± SEM. Statistical analyses were performed using Excel and GraphPad 9 software (Prism).

## Results

### Expression of cytokines and receptors of the IL-1 family by skin cells

**Figure 1:**
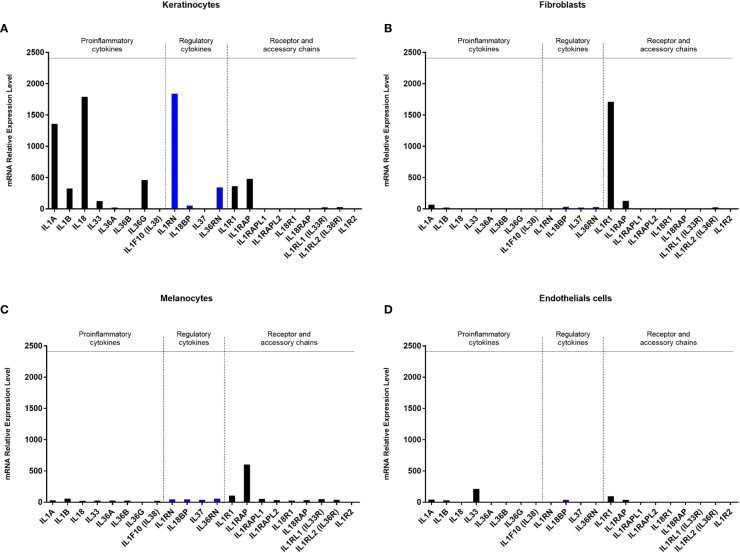
Gene expression profiles of proinflammatory cytokines, regulatory cytokines and receptors of the IL-1 family by different skin cell populations. The expression of the IL-1 family members and their receptors was analyzed at the transcriptional level by Affymetrix analysis (hU219 array) after mRNA extraction of primary cultures of epidermal keratinocytes **(A)**, dermal fibroblasts **(B)** melanocytes **(C)** and dermal microvascular endothelial cells **(D)**, all isolated from human skin and cultivated in healthy conditions. Averaged data obtained from 2 independent cultures for each cell type are presented in relative expression of the gene of interest.

Before modelling the epidermal lesion at the cellular level, we first determined the mRNA expression profiles of the IL-1 family members and their receptors by the most abundant skin cells, including primary epidermal keratinocytes, dermal fibroblasts, melanocytes, and dermal microvascular endothelial cells isolated from human skin, all cultivated *in vitro* in physiological conditions (**Figures 1A–D**). As expected, keratinocytes were found to express IL-1α, IL-1β, IL-18 and IL36G at high levels compared to the other skin cells studied in which the levels of the proinflammatory cytokines were low or undetectable. Keratinocytes were also the only skin cells to express significant levels of the regulatory/antagonist cytokines IL-1RA (IL-1RN) and IL-36RN. By contrast, dermal fibroblasts represented by far the cell population expressing the highest level of the IL-1R1 chain together with the IL-1RAP chain (also named IL-RAcP or IL-1R3) to form the functional receptor that binds both IL-1α and IL-1β (**Figures 1A–D**). The expression of receptors of the IL-1 family in the other cell types was detectable but at much lower levels. Of note, none of the analyzed skin cells expressed significant levels of IL-1R2, a decoy receptor for IL-1. For comparison, we also analyzed the expression of IL-1 family members in immune blood cells (PBMC, CD4^+^ T cells, monocytes and polymorphonuclear cells (PMN), **Supplementary Figure 1**). Among proinflammatory cytokines, all blood cell types expressed significant amounts of IL-1β and IL-1α, although at a lesser extent than keratinocytes. Most blood cells also expressed regulatory/antagonist cytokines and significant levels of IL-1R1 receptor (together with IL-1RAP), but most strikingly the expression profile of blood cells clearly differed from skin cells due to the significant expression levels of the decoy receptor IL1-R2 (especially in monocytes and PMN).

In conclusion, this transcriptional analysis confirmed that epidermal keratinocytes are the main cells expressing the proinflammatory cytokines IL-1α and IL-1β in the skin and suggests that, among skin cells, dermal fibroblasts could be the strongest responders to IL-1 after epidermis injury in aseptic conditions.

### Fibroblasts are the most responsive skin cells to lesioned keratinocytes

**Figure 2:**
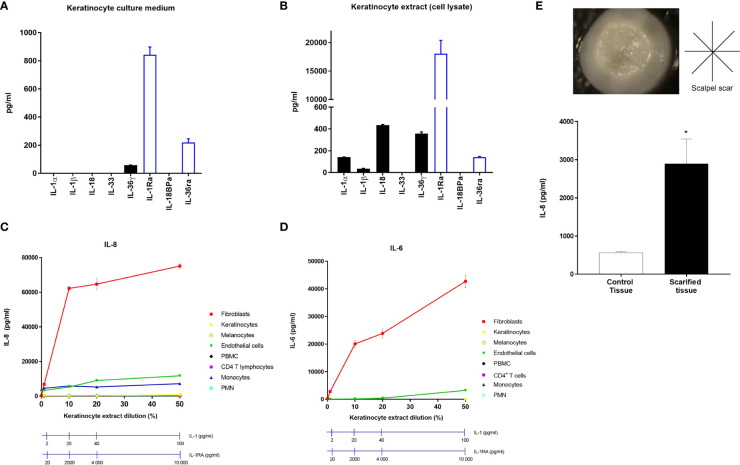
Dermal fibroblasts are stimulated by keratinocyte extracts. Concentrations of the IL-1 family members, IL-1α, IL-1β, IL-18, IL-33, IL-36γ, IL-1RA, IL-18BP and IL-36RA were measured by ELISA in culture medium of keratinocytes cultivated in healthy conditions **(A)** or in keratinocyte extracts after cell lysis **(B)**. Several cell types including fibroblasts, keratinocytes, melanocytes, endothelial cells, PBMC, CD4 T cells, monocytes and PMN were stimulated with different dilutions of keratinocyte extracts. IL-8 **(C)** and IL-6 **(D)** concentrations were measured by ELISA in culture medium of stimulated fibroblasts. Concentrations of IL-1 and IL-1RA were measured for each keratinocyte extract dilution and were indicated above **(C, D)**. Reconstituted human skins were scarified with a scalpel under sterile conditions according to the indicated cross-shaped scheme **(E)**. Scars are observable on the representative image of the reconstituted human skin. IL-8 concentrations were measured in the underlying culture medium, 48 h after scarification or in non-lesioned control tissue. Data are representative of three independent experiments done in triplicate. *P < 0.05, using the Mann & Whitney’s test for comparison between groups.

To test the hypothesis that dermal fibroblasts could be the strongest responder cells to IL-1, we compared *in vitro* the response of the different populations of cutaneous and immune cells after exposure to keratinocyte extracts. As keratinocytes do not release the proinflammatory cytokines IL-1α and IL-1β in homeostatic conditions (**Figure 2A**), we used keratinocyte lysates for cell stimulation, in which elevated concentrations of IL-1α, IL-1β, IL-18 and IL-36γ were detected (**Figure 2B**). It is noteworthy that significant concentrations of the secreted regulatory cytokines IL-1RA and IL-36RA were found in culture medium of keratinocytes but even stronger concentrations of IL-1RA were measured in keratinocyte extracts, ~100-fold higher than IL-1α concentrations (**Figures 2A, B**).

The response of individual skin and immune cells was analyzed by measuring IL-8 (**Figure 2C**) and IL-6 (**Figure 2D**) release in culture medium after stimulation with increasing doses of keratinocyte extracts. Our results clearly showed that dermal fibroblasts are the most responsive cells as demonstrated by the massive secretion of IL-8 and IL-6 in the presence of keratinocyte extracts at a dilution of 10% only, corresponding to an IL-1α/β concentration of 20 pg/ml, despite the presence of IL-1RA at a concentration of 2000 pg/ml. Only endothelial cells and monocytes were found to release detectable but much lower amounts of IL-8 and IL-6 after exposure to higher concentrations of keratinocyte extracts (dilutions between 20% to 50%, corresponding to IL-1 concentrations ranging from 40 to 100 pg/ml and IL-1RA concentrations ranging from 4 to 10 ng/ml). This experiment showed that, except fibroblasts, most of the skin cells studied, including keratinocytes, did not respond to keratinocyte extracts.

To model epidermal lesion *in vitro* in a more complex manner, we used a three-dimensional model of human reconstituted skin (RHS), composed of a stratified epidermis developed from human primary keratinocytes cultivated at the air-liquid interface, at the surface of a dermal equivalent (human primary dermal fibroblasts embedded in a collagen lattice). RHS were superficially scarified with a sterile scalpel to induce epidermis injury and IL-8 concentrations were measured in the underlying culture medium 48 h after scarification (**Figure 2E**). The epidermal lesion induced a significant release of IL-8 by comparison to the non-lesioned control tissue.

Together, these findings demonstrate that dermal fibroblasts are the most responsive skin cells to epidermal injury in an aseptic context.

### IL-1 released by keratinocytes is the most potent inducer of the fibroblast response

**Figure 3:**
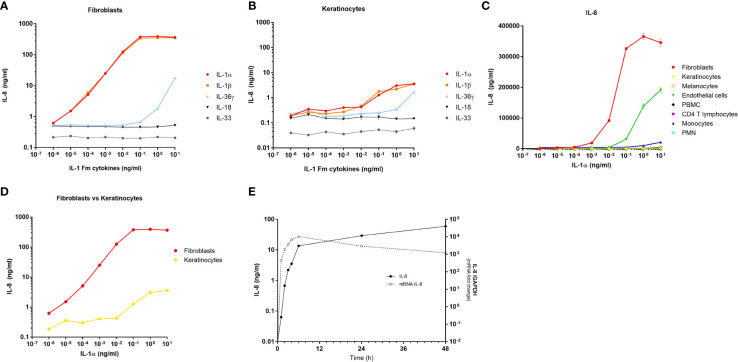
Dermal fibroblasts are the best responder skin cells to IL-1α/β. Dermal fibroblasts **(A)** and epidermal keratinocytes **(B)** were stimulated for 24 h with increasing concentrations of IL-1 family members including, IL-1α, IL-1β, IL-36γ, IL-18 and IL-33. The fibroblast response to cytokines was evaluated by measuring IL-8 concentrations in culture medium by ELISA. Several cell types including fibroblasts, keratinocytes, melanocytes, endothelial cells, PBMC, CD4 T cells, monocytes and PMN were stimulated for 24 h with increasing concentrations of IL-1α before measuring IL-8 concentrations in culture medium by ELISA **(C)**. Comparison between fibroblast and keratinocyte responses to increasing concentrations of IL-1α by IL-8 measurements in culture medium after 24 h of stimulation **(D)**. Kinetics of IL-8 mRNA expression and IL-8 protein release by fibroblasts stimulated with 100 pg/ml of IL-1α **(E)**. IL-8 mRNA expression was determined by RT-qPCR and data were presented as relative expression to the housekeeping gene GAPDH. IL-8 dosages in culture medium were performed by ELISA.

To confirm that the response of dermal fibroblasts to keratinocyte extracts is mainly mediated by IL-1, we compared the stimulating effect of single recombinant cytokines on fibroblasts, including IL-1α, IL-1β, IL-36G, IL-18 and IL-33 (**Figure 3A**). Among these IL-1 family members, IL-1α and IL-1β were the most potent cytokines to induce IL-8 release by fibroblasts, with an increasing effect at concentrations ranging from 10^-5^ to 10^-1^ ng/ml for both cytokines. The stimulating effect of IL-1α/IL-1β reached a plateau from 10^-1^ ng/ml with a massive induction of IL-8 release which remained constant at higher concentrations of IL-1α/IL-1β (IL-8 concentrations above 100 ng/ml). IL-36G was also able to stimulate IL-8 secretion but at higher concentrations than IL-1 (above 1 ng/ml), while IL-18 and IL-33 failed to stimulate fibroblasts. In comparison, a much weaker effect of IL-1α and IL-1β was observed in human primary keratinocytes on IL-8 secretion, with stimulating concentrations observed only above 10^-1^ ng/ml (**Figure 3B**). Also, the highest concentrations observed for IL-8 release by keratinocytes never exceeded 5 ng/ml.

Considering that IL-1α and IL-1β are the most powerful cytokines of the IL-1 family and that both members have a comparable and redundant activity by binding to the shared ILR1/IL-1RAP receptor, we compared the response of the different cell populations stimulated with IL-1α only, by analyzing IL-8 release in culture medium. We confirmed that dermal fibroblasts were the most responsive cells to IL-1α, followed by endothelial cells, while the other cells were non-responders or low-responders (**Figure 3C**). This analysis was performed on several cultures of dermal fibroblasts isolated from different anatomical sites (breast, abdomen, ear, foreskin) and from patients with different ages (from 7 months to 51 years) to demonstrate that all fibroblasts tested have the same capacity to respond to IL-1α (**Supplementary Figure 2**). The comparison between fibroblasts and keratinocytes responses reveals that keratinocytes must be exposed to ~10.000-fold higher concentrations of IL-1α to secrete similar amounts of IL-8 by fibroblasts (**Figure 3D**). In addition, starting from the concentration of 10^-3^ ng/ml of IL-1α, the response of fibroblasts is already ~100-fold more elevated than those of keratinocytes. Finally, by measuring at several time points both IL-8 expression at the transcriptional level and IL-8 release at the protein level, we showed that the fibroblast response to IL-1α exposure is very fast, with a maximum effect observed in few hours only, including at the protein level (**Figure 3E**).

**Figure 4:**
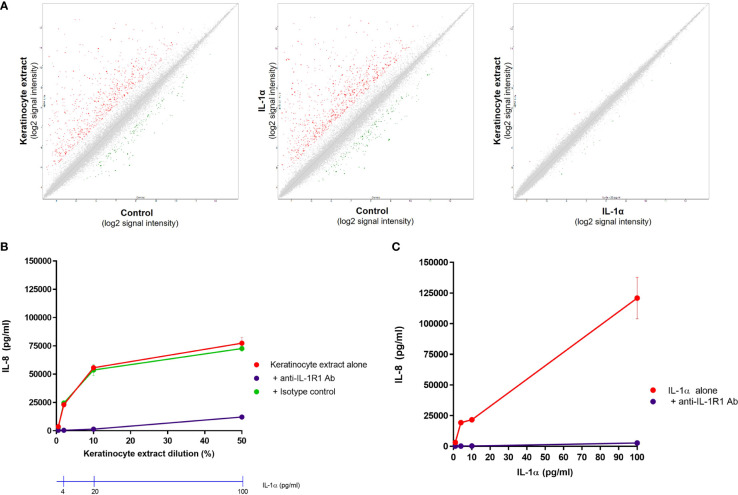
The response of dermal fibroblasts to keratinocyte extracts is mainly mediated by IL-1α/β. Complementary Affymetrix microarray experiments (hU219 array) were performed to analyze the expression of 36000 transcripts (corresponding to ~20000 genes) by normal dermal fibroblasts (control) and fibroblasts stimulated either by keratinocyte extracts (tested at a dilution of 10%, containing ~20 pg/ml of IL-1α) or IL-1α (tested at 20 pg/ml). Comparisons of the transcriptional profiles between two conditions were shown, with up-regulated genes shown in red and down-regulated genes in green **(A)**. Dermal fibroblasts were stimulated by different dilutions of keratinocyte extracts **(B)** or increasing concentrations of IL-1α **(C)** in the presence of an anti-IL-1R1 blocking antibody or an isotype control. Corresponding concentrations of IL-1α in keratinocyte extracts were also indicated **(B)**.

We next investigated whether the effect of keratinocyte extracts on dermal fibroblasts is mediated only by IL-1α/β or also by other potential proinflammatory mediators. We first compared the transcriptomic profiles of unstimulated and stimulated fibroblasts by analyzing the expression of ~30.000 transcripts by microarray experiments. Compared to control cells, fibroblasts stimulated by keratinocyte extracts or by recombinant IL-1α presented highly similar profiles of gene regulation, explaining why both stimulated conditions (e.g., keratinocyte extracts vs IL-1α) showed an overlaying profile (**Figure 4A**). Interestingly, among the top twenty genes whose expressions were the most upregulated, eight were coding for chemokines including many neutrophil-attracting chemokines (**Supplementary Table 1**). We further stimulated fibroblasts with increasing concentrations of keratinocyte extracts (**Figure 4B**) or recombinant IL-1α (**Figure 4C**) in the presence of a blocking anti-IL-1R1 antibody or an isotype control. Since the anti-IL-1R1 antibody blocked efficiently IL-8 secretion by fibroblasts after keratinocyte extracts stimulation, we can state that the biological effect of keratinocyte extracts was specifically mediated by IL-1α/β.

### Fibroblasts are very sensitive to low concentrations of IL-1 even in the presence of high concentrations of IL-1Ra

**Figure 5:**
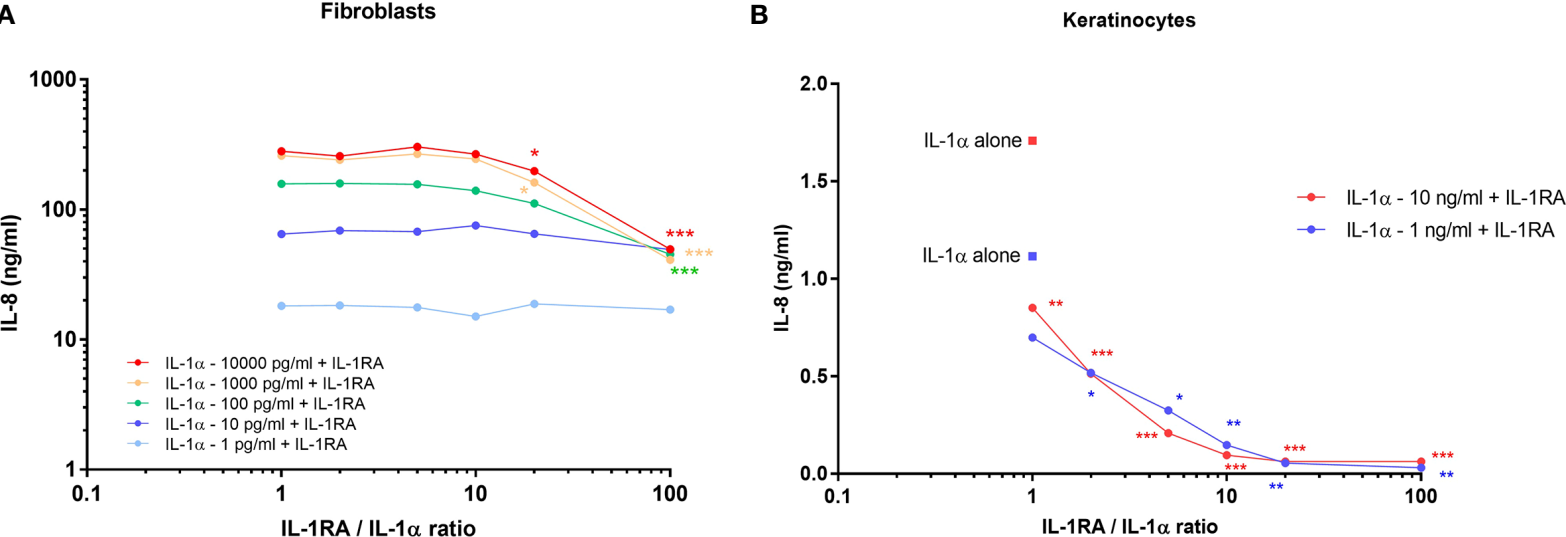
Dermal fibroblasts are highly sensitive to IL-1α despite the presence of elevated concentrations of IL-1RA. Dermal Fibroblasts **(A)** and keratinocytes **(B)** were stimulated for 24 h with increasing concentrations of IL-1α in presence of different concentrations of IL-1RA at IL-1RA/IL-1α ratios ranging from 1 to 100. The concentrations of IL-8 released by stimulated cells were measured by ELISA in culture medium. *P < 0.05, **P < 0.01, ***P < 0.001 using the Mann & Whitney’s test.

The analysis of the composition of keratinocyte extracts realized above revealed high concentrations of the antagonist IL-1RA, which is known to bind to the IL-1α/β receptor and block its activity. However, although the concentrations of IL-1RA were 100-fold more elevated than IL-1 concentrations in keratinocyte extracts, we showed that the activity of IL-1α/β on fibroblastic cells was not abolished. This interesting finding led us to evaluate *in vitro* the responsiveness of dermal fibroblasts to IL-1α exposed to increasing doses of IL-1RA. Thus, we analyzed IL-8 release by fibroblasts, and keratinocytes for comparison, after stimulation with different concentrations of IL-1α (from 1 pg/ml to 10 ng/ml) in the presence of IL-1RA at different ratios (1, 2, 5, 10, 20 and 100) (**Figure 5A**). Up to an IL-1RA/IL-1α ratio of 10, the activity of IL-1α remained unaffected. The activity of IL-1α was significantly affected at all tested concentrations (except the lowest) only when the concentration of IL-1RA reached 100-fold the concentration of IL-1α (IL-1RA/IL-1α ratio of 100 as in the keratinocyte extracts), but it should be noted that the activity of IL-1α still remained elevated (similar to a 10 pg/ml IL-1α stimulation without IL-1RA with an IL-8 secretion above 10 ng/ml). By contrast, when keratinocytes were stimulated with IL-1α at concentrations of 1 and 10 ng/ml, a blocking effect of IL-1RA was rapidly observed already at an IL-1RA/IL-1α ratio of 1. For an IL-1RA/IL-1α ratio ≥ 10, the stimulating effect of IL-1α on IL-8 secretion was almost abolished (**Figure 5B**). Altogether, these results confirmed the high responsiveness of fibroblasts to IL-1α, even in presence of elevated concentrations of the antagonist IL-1RA, a feature which was not observed in keratinocytes.

### IL-1-activated fibroblasts have the capacity to attract neutrophils both *in vitro* and *in vivo*

**Figure 6:**
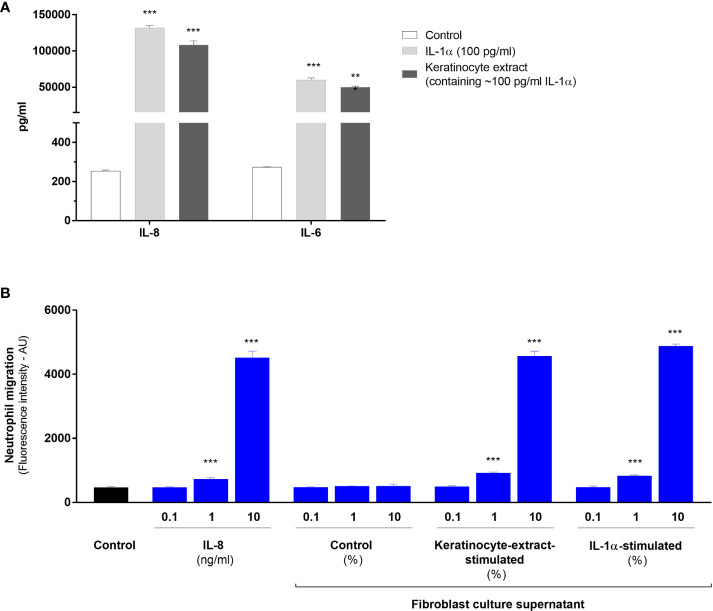
IL-1-activated fibroblasts have the capacity to attract neutrophils *in vitro*. Concentrations of IL-8 and IL-6 were determined by ELISA in culture supernatants from dermal fibroblasts stimulated with IL-1α at a concentration of 100 pg/ml or a half-diluted keratinocyte extract containing IL-1α at a similar concentration of 100 pg/ml **(A)**. Analysis of cell migration determined by Boyden chamber assays **(B)**. Polymorphonuclear cells were seeded in the upper compartment for migration and the lower compartment contained either culture medium (control), or different concentrations of IL-8, control supernatant of unstimulated fibroblasts or supernatants from fibroblasts stimulated either with IL-1α (100 pg/ml) or a half-diluted keratinocyte extract. Data were obtained from 2 independent experiments done in triplicate and presented as mean fluorescence intensity ± SEM. ***P < 0.001 using the Mann & Whitney’s test.

Finally, we carried out functional experiments to demonstrate that IL-1-activated fibroblasts have the capacity to elicit an inflammatory response by attracting immune cells on the site of injury after aseptic epidermal lesion. As previously shown, fibroblasts responded to IL-1α by overexpressing numerous neutrophil-attracting chemokines, particularly IL-8 which has been used constantly as a read-out parameter for our *in vitro* studies. To determine the capacity of activated fibroblasts to attract neutrophils, we firstly performed *in vitro* cell migration assays using Boyden chambers. PMN were seeded in the upper compartment, while the lower compartment contained different dilutions of culture supernatants of unstimulated fibroblasts (control supernatant), supernatants from fibroblasts stimulated either with keratinocyte extracts or IL-1α, or culture medium containing different concentrations of IL-8 (positive control). Culture supernatants of fibroblasts stimulated with 100 pg/ml of IL-1α or keratinocyte extract (containing approximatively 100 pg/ml of IL-1α), contained similar concentrations of IL-8 (~ 100 ng/ml), IL-6 (~ 50 ng/ml) (**Figure 6A**) and the metalloproteinase MMP- also involved in cell migration (~ 40 ng/ml, data not shown). Boyden chamber migration assays showed the capacity of 10%-diluted supernatants from stimulated-fibroblasts to attract neutrophils in the lower compartment at the same level as IL-8 at a concentration of 10 ng/ml (**Figure 6B**). A weak but significant cell migration could be observed when supernatants of stimulated fibroblast were used at a 1% dilution or IL-8 at a concentration of 1 ng/ml.

**Figure 7:**
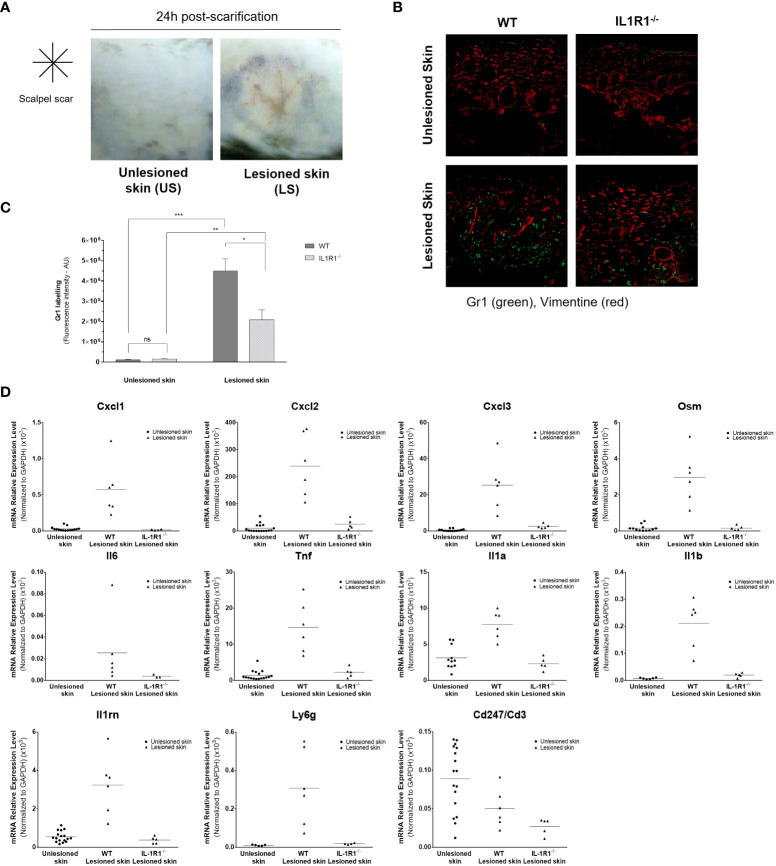
Aseptic epidermal lesion induces skin infiltration of neutrophils in an IL-1-dependent manner. The epidermis of the back skin of WT and IL-1R1-deficient (IL-1R1^-/-^) mice was scarified in aseptic conditions using a sterile scalpel. Representative pictures of mouse unlesioned skin and the lesioned back skin of mice 24 h after a cross-shaped scarification **(A)**. Immunofluorescence staining of Gr1-positive neutrophils (in green) and the mesenchymal marker vimentin (in red), in skin sections of unlesioned and lesioned skins of WT and IL-1R1-deficient mice **(B)**. Quantitative analysis of Gr1-specific fluorescent labelling in unlesioned and lesioned skin sections of WT and IL-1R1-deficient mice **(C)**. Transcriptional analysis by RT-qPCR after mRNA extraction from lesioned back skins of WT and IL-1R1-KO mice and unlesioned skins (both from WT and IL-1R1-KO mice) 24 h after scarification **(D)**. The expression of *CXCL1*, *CXCL2*, CXCL3, *OSM, IL-6, TNFα, IL-1α, IL-1β, IL-1RA, Ly6G *and* CD3ζ (CD247)* was analyzed and expressed as mean ± SEM relative expression to the housekeeping gene *Gapdh*. *P < 0.05, **P < 0.01, ***P < 0.001 using the Mann & Whitney’s test.

To confirm these results *in vivo* and to demonstrate the major role of IL-1 in aseptic skin inflammation, we modelled the epidermal lesion by scarifying superficially the epidermis of the depilated back skin of C57BL6/J wild-type (WT) mice or IL-1R1-deficient mice using a sterile scalpel (**Figure 7A**). In these transgenic mice, we previously verified *in vitro* that murine primary keratinocytes were unable to respond to IL-1 (**Supplemental Figure 3**). Twenty-four hours after scarification, the lesioned and unlesioned skins were collected and the detection of neutrophils was analyzed in skin sections by immunohistofluorescence using Gr1 antibody. A strong infiltration of Gr1-positive cells could be observed in the dermis of the lesioned skins of WT mice (**Figures 7B, C**); however, in the lesioned skin of IL-1R1-deficient mice, the infiltration of neutrophils was significantly lower and almost absent in unlesioned skins in both strains of mice. A transcriptional analysis of inflammatory mediators was also performed on unlesioned and lesioned skins of WT and IL-1R1-deficient mice 24 h after scarification (**Figure 7D**). While an overexpression of neutrophil-attracting chemokines (e.g., CXCL-1/2/3), inflammatory cytokines (e.g., oncostatin M, IL-6, TNFα, IL-1α, IL-1β) and the anti-inflammatory IL-1RA could be observed in the lesioned skin of WT mice, these overexpressions were almost abolished in the lesioned skins of IL-1R1-deficient mice at similar levels to those of unlesioned skins (p < 0.01 using the Mann & Whitney test for all genes tested, excepted for IL-6 (p < 0.05), and CD3 (no significant difference). Consistently with our observation by immunohistofluorescence, the expression of the Gr1 marker (Ly-6G) was also found reduced in the lesioned skin of IL-1R1-deficient mice compared to WT mice. By contrast, no difference in CD3 expression could be observed suggesting that the IL-1-mediated skin inflammation is not associated with T cells and adaptative immunity. Altogether, these *in vivo* results reinforce our *in vitro* data on the major contribution of IL-1α/β in sterile inflammation by attracting neutrophils to the site of epidermis injury.

## Discussion

The role of IL-1 in inflammatory processes has been described for a long time and has led to substantial body of literature (21). In the context of sterile inflammation after tissue injury on which we have focused, Chen and colleagues reported that dying lymphoblastic cells have the capacity to drive an acute neutrophilic and monocytic inflammatory response when they were injected intraperitoneally to mice (16). They demonstrated that the neutrophil response was dependent on IL-1α and required IL-1R expression on non-bone marrow-derived cells, suggesting the involvement of IL-1-sensitive cells playing an intermediate role in the inflammatory cascade that remain to be determined. In a model of hypoxia-induced cell death, IL-1α and IL-1β were both shown to be released by dying keratinocytes to trigger sterile inflammation along with neutrophil and macrophage recruitment (15). However, the nature of the potential IL-1-responder cells was not determined in this study. Therefore, we have investigated the role of IL-1 released by keratinocytes after epidermal injury to provide new insights on the molecular and cellular mechanisms of sterile skin inflammation and to identify potential important relay cells in this process. We first analyzed the mRNA expression profiles of cytokines and receptors of the IL-1 family in different skin cell populations and in cells of hematopoietic origin for comparison. Unsurprisingly, we found that keratinocytes are the main source of IL-1α and IL-1β in the skin, as well as IL-18, IL-33 and IL-36γ, as previously described (5, 9–11). More interestingly, we show that dermal fibroblasts have the highest expression level of the IL-1R1 chain compared to keratinocytes, melanocytes and skin-isolated endothelial cells. Our results are in accordance with a previous study in which the expression of the IL-1R1 chain was analyzed in PBMC and skin-isolated cells (22). This expression level in fibroblasts was comparable to those of neutrophils, suggesting that these cells might be a good target for IL-1α and IL-1β released by injured keratinocytes. To test this hypothesis, we exposed the different skin cells to protein extracts of lysed keratinocytes which contained significant concentrations of IL-1α, IL-1β, IL-18, IL-33 and IL-36γ, with IL-1α concentrations similar to those measured in supernatants of hypoxia-induced dying keratinocytes (15). Although these cytokine concentrations are elevated and certainly do not reflect *in vivo* conditions of physiological and/or pathological keratinocyte death, we exposed cells to different dilutions of keratinocyte extracts and demonstrated that, among these skin cells, dermal fibroblasts were by far the most responsive cells by releasing rapidly the neutrophil-attracting chemokine IL-8 and the proinflammatory cytokine IL-6. Using a more sophisticated *in vitro* model of injured reconstituted human skin, the superficial lesion of epidermis also resulted in IL-8 release. As injured keratinocytes could potentially release other danger-associated molecular patterns or alarmins in addition to cytokines of the IL-1 family (4), we addressed the question of the real contribution of IL-1α and IL-1β to dermal fibroblast activation. Both cytokines have the highest capacity to stimulate dermal fibroblasts at very low concentrations (from 0.01 pg/ml). When fibroblasts were stimulated with keratinocyte extracts or recombinant cytokines in the presence of anti-IL-1R1 neutralizing antibodies, fibroblast activation was strongly abolished, demonstrating that sterile inflammation following epidermal injury is mainly driven by IL-1α and IL-1β. Of note, skin-derived endothelial cells were identified as the second-best responder cells but to higher concentrations of IL-1 and at a lower intensity than fibroblasts. The capacity of human dermal microvascular endothelial cells was already investigated thirty years ago by Kristensen et al., showing that endothelial cells produced very high, fibroblasts and monocytes intermediate, and keratinocytes low amounts of IL-8 mRNA using a dot blot mRNA hybridization method (23). Using more sensitive and specific techniques at the protein level, we can state that fibroblasts have a higher capacity to respond to IL-1 than endothelial cells.

The contribution of fibroblasts as relay cells in the cascade of sterile inflammation after tissue injury and their capacity to respond to IL-1 has always been neglected or underestimated, most studies focusing on the activity of IL-1 on PBMCs, in particular on neutrophils and monocytes whose activation is essential for tissue repair (15, 16). If keratinocyte-fibroblast interactions have been investigated in wound healing processes, the role of IL-1 released by injured keratinocytes was focused on the regulation of the TGF-β-induced transformation of dermal fibroblasts into myofibroblasts during wound contraction (24). In fact, most studies on dermal fibroblasts as a target of IL-1 were carried out in the context of chronic skin inflammatory diseases. In systemic sclerosis within the fibroblasts play a central role in the pathophysiology, the interplay between pathological keratinocytes and the underlying fibroblasts involving IL-1α was described to be important in the fibrotic process (25). IL-1β has been also found highly expressed in hidradenitis suppurativa skin lesions and targets fibroblasts to modulate gene expression of molecules involved in remodeling of the extracellular matrix, chemokines, adhesion molecules or cytokines (22). Interestingly, in this study, they identified, like us, dermal fibroblasts as the skin-resident cell type harboring the highest number of gene regulation after IL-1β stimulation.

Surprisingly, our results showed the capacity of dermal fibroblasts to respond to low concentrations of IL-1α and IL-1β in the presence of elevated concentrations of the antagonist IL-1RA, although it is known to bind to IL-1R in a competitive manner with IL-1α and IL-1β. Four isoforms of IL-1RA exist, one secreted form and three intracellular forms which can be released by dying cells and compete extracellularly for IL-1R1 binding in a manner similar to the secreted IL-1RA (26). Although our method for IL-1RA dosage do not allow to discriminate between these isoforms, we could detect 100-fold higher concentrations of IL-1RA compared to IL-1α and IL-1β concentrations in our keratinocytes extracts without affecting its capacity to strongly stimulate fibroblasts, whereas the response of keratinocyte to IL-1 was almost abolished in the presence of 10-fold higher concentrations of IL-1RA. This finding might be explained by the difference between IL-1R1 expression levels between both cell types. Only a small fraction of the cell surface heterodimeric IL-1R1/IL1-RAP receptor needs to be bound by IL-1 to induce an activation signal in fibroblasts, even if the majority of the IL-1R1 chains on fibroblast surface are mobilized for IL-1RA binding. Our observation is in agreement with a previous study showing that a 10^4^-fold excess of IL1-RA over the level of IL-1α was required to significantly reduce the activity of the agonist cytokine on a murine fibroblastic cell line (27). As this particularity concerns fibroblasts only, this might suggest that IL1-RA released together with IL-1 should protect other skin-resident cells from activation and limit the local inflammatory response.

Finally, we show that dermal fibroblasts are important relay cells for neutrophil recruitment to the site of tissue injury through their capacity to release neutrophil-attracting chemokines. In response to IL-1 or keratinocyte extract exposure, CXCL-1/2/3 and IL-8 were found among the most up-regulated genes by stimulated fibroblasts. Our *in vitro* cell migration assay demonstrated the capacity of culture supernatant of stimulated fibroblasts to attract neutrophils efficiently. As well, our *in vivo* experiments of superficial epidermal lesions on the back skin of IL-1R1-deficient mice confirmed the crucial role of IL-1 in the development of a sterile inflammation and the recruitment of neutrophils in the underlying dermis. However, since neutrophil infiltration was reduced, but not abolished, in the lesioned skin of IL-1R1-deficient mice, this finding suggests that IL-1 is not the only relevant cytokine responsible for neutrophil recruitment and that other proinflammatory mediators may also be implicated. For instance, IL-18 and IL-36 were also found at high concentrations in our keratinocyte extracts. While IL-18 is mostly associated with macrophage recruitment *in vivo* (15), IL-36 has been reported to attract neutrophils in the skin (28). Altogether, our results are in accordance with the previous studies reporting the contribution of IL-1 in sterile inflammation induced by dying cells (15, 16).

In conclusion, we demonstrate that dermal fibroblasts are key relay cells in the cascade of sterile inflammation induced by epidermis injury. They are potent responder cells of IL-1 released by injured keratinocytes, extremely sensitive to low concentrations of IL-1 even in the presence of elevated concentrations of IL-1RA, and contribute to the recruitment of neutrophils involved in tissue repair *via* the secretion of relevant chemokines. Mostly underestimated in the inflammatory process, more attention should be paid to dermal fibroblasts considering their high sensitivity to IL-1, particularly in strategies which consist in regulating IL-1 activity through the use of IL-1RA or neutralizing anti-IL-1R1 antibodies.

## Data availability statement

The datasets presented in this study can be found in online repositories. The name of the repository and accession number can be found below: NCBI Gene Expression Omnibus; GSE111191.

## Ethics statement

Experimental procedures were approved by French Government’s ethical and animal experiment regulations and were submitted to the ethics committee for animal Experimentation of “CNRS Campus Orleans” (CCO) and approved under APAFIS#30196-2019021818223038.

## Author contributions

Conceived and designed the experiments: SC-D, F-XB, J-CL, FM and J-FJ. Performed the experiments: SC-D, NP, JG, SC-C, VH, J-FJ. Analyzed the data: SC-D, NP, JG, SC-C, F-XB, VH, J-FJ. Contributed reagents/materials/analysis tools/animals: BR. Wrote the paper: SC-D, VH, J-FJ. All authors contributed to the article and approved the submitted version.

## Funding

This work was supported by grants from Qima-Bioalternatives, the University of Poitiers and CNRS, University of Orleans, ‘Fondation pour la Recherche Médicale’ (EQU202003010405) and European funding in Region Centre-Val de Loire (FEDER N° EX010381). Publication charges were supported by the ‘Direction de la Recherche et de l’Innovation’ of Poitiers Hospital (CHU de Poitiers).

## Acknowledgments

We thank Stéphanie Rose (INEM, CNRS, UMR 7355, Orléans, France) for technical help for animal experimentation, and Sarah Collober (Qima-Bioalternatives) for formatting the figures and the tables of the article.

## Conflict of interest

Authors SC-D, NP, JG, SC-C, F-XB and VHA were employed by Qima-Bioalternatives (Qima Life Sciences).

The remaining authors declare that the research was conducted in the absence of any commercial or financial relationships that could be construed as a potential conflict of interest.

The authors declare that this study received funding from Qima-Bioalternatives. The funder had the following involvement with the study: design, collection, analysis, interpretation of data, the writing of this article and the decision to submit it for publication.

## Publisher’s note

All claims expressed in this article are solely those of the authors and do not necessarily represent those of their affiliated organizations, or those of the publisher, the editors and the reviewers. Any product that may be evaluated in this article, or claim that may be made by its manufacturer, is not guaranteed or endorsed by the publisher.

## Abbreviations

HMVEC, Human dermal microvascular endothelial cells; NHDF, Normal human dermal fibroblasts; NHEK, Normal human epidermal keratinocytes; NHEM, Normal human epidermal melanocytes; PBMC, Peripheral blood mononuclear cells; PMN, Polymorphonuclear cells; RHS, Reconstituted human skin; WT, Wild-type.
