# Supplementary material for: Dermal fibroblasts are the key sensors of aseptic skin inflammation through interleukin 1 release by lesioned keratinocytes
Source: Front Immunol. 2022 Oct 3;13:984045. doi: 10.3389/fimmu.2022.984045 (PMC9576869; doi:10.3389/fimmu.2022.984045)
Supplement: Supplementary file 1 [file DataSheet_1.docx]

***Supplementary Material***

1. **Supplementary material and methods**

**Cell culture**

Primary normal human epidermal keratinocytes (NHEK) and normal human dermal fibroblasts (NFDF) were obtained from surgical samples of healthy skin collected by the Plastic Surgery Unit of Poitiers Hospital. Primary cells were isolated as previously described ([1](#_ENREF_1), [2](#_ENREF_2)). Primary human melanocytes (NHEM) were isolated from healthy children’s foreskin as previously described ([3](#_ENREF_3)). Primary Human dermal Microvascular Endothelial Cells (HMVEC), isolated from the dermis of juvenile foreskin and adult skin, were purchased from Promocell. All cells and tissues were maintained in a humidified atmosphere 5% CO_2_ at 37°C.

NHEK were cultured in complete keratinocyte serum-free medium (SFM) supplemented with bovine pituitary extract (25 μg/ml), recombinant epidermal growth factor (EGF) (0,25 ng/ml) and gentamycin (25 μg/ml). NHDF were cultured in DMEM medium supplemented with 2 mM L-glutamine, 10% inactivated Fetal Bovine Serum (FBS), and 1% Penicillin/Streptomycin (P/S). For cultures dedicated to gene expression analysis, the percentage of FBS was decreased to 1%. NHEM were cultured in M254 medium supplemented with PMA-free human melanocyte growth supplement 2 (HMGS-2), 1% Penicillin/Streptomycin (P/S) and 1% gentamycine (25 μg/ml). Twenty-four hours after seeding, the culture medium was replaced by an assay medium containing no hydrocortisone. HMVEC were grown in complete supplemented Endothelial cell growth medium 2 (Promocell). Twenty-four hours after seeding, the culture medium was replaced by an assay medium containing no hydrocortisone and no serum. All blood cells were cultured in RPMI 1640 medium supplemented with 2 mM L-glutamine, 10% inactivated Fetal Bovine Serum (FBS), and 1% Penicillin/Streptomycin (P/S).

All culture media and supplements listed above were purchased from Thermo Fischer scientific (GibcoTM), excepted for HMVEC.

NHEK were used at passage 3, whereas NHDF, NHEM and HMVEC were used at passage 8. All blood cells were used immediately after isolation.

1. Boniface K, Lecron JC, Bernard FX, Dagregorio G, Guillet G, Nau F, et al. Keratinocytes as Targets for Interleukin-10-Related Cytokines: A Putative Role in the Pathogenesis of Psoriasis. *Eur Cytokine Netw* (2005) 16(4):309-19. Epub 2006/02/09.

2. Huguier V, Giot JP, Simonneau M, Levillain P, Charreau S, Garcia M, et al. Oncostatin M Exerts a Protective Effect against Excessive Scarring by Counteracting the Inductive Effect of Tgfbeta1 on Fibrosis Markers. *Sci Rep* (2019) 9(1):2113. Epub 2019/02/16. doi: 10.1038/s41598-019-38572-0

10.1038/s41598-019-38572-0 [pii].

3. Gontier E, Cario-Andre M, Lepreux S, Vergnes P, Bizik J, Surleve-Bazeille JE, et al. Dermal Nevus Cells from Congenital Nevi Cannot Penetrate the Dermis in Skin Reconstructs. *Pigment Cell Res* (2002) 15(1):41-8. Epub 2002/02/12. doi: 10.1034/j.1600-0749.2002.00065.x.

1. **Supplementary tables**

**Table S1. Primer sequences used in this study.**

| **Gene name** | **Abbreviation** | **Forward (5’ →3’)** | **Reverse (5’ →3’)** |
| --- | --- | --- | --- |
| [Glyceraldehyde-3-phosphate dehydrogenase](http://www.ncbi.nlm.nih.gov/gene/14433) | Gapdh | AACTTTGGCATTGTGGAAGG | ACACATTGGGGGTAGGAACA |
| [Interleukin 1 alpha](http://www.ncbi.nlm.nih.gov/gene/16175) | Il1a | GCAGCAGGGTTTTCTAGGTG | CTTTAAGGACGGGAGGGAGA |
| [Interleukin 1 beta](http://www.ncbi.nlm.nih.gov/gene/16176) | Il1b | GCTCAGGGTCACAAGAAACC | CAAGTGCAAGGCTATGACCA |
| [Interleukin 1 receptor antagonist](http://www.ncbi.nlm.nih.gov/gene/16181) | Il1rn | TGTGCCAAGTCTGGAGATGA | AGTGTTGTGCAGAGGAACCA |
| [Interleukin 1 receptor, type I](http://www.ncbi.nlm.nih.gov/gene/16177) | Il1r1 | GAATGACCCTGGCTTGTGTT | TGTGCTCTTCAGCCACATTC |
| Lymphocyte antigen 6 complex, locus G | Ly6g | GATGGATTTTGCGTTGCTCT | AGGACTGAAACCAGGCTGAA |
| [CD247 antigen](http://www.ncbi.nlm.nih.gov/gene/12503) | Cd247 | CTGCTACACACCAGCCTCAA | GCCTTGGCCTTCCTATTCTT |
| [Chemokine (C-X-C motif) ligand 1](http://www.ncbi.nlm.nih.gov/gene/14825) | Cxcl1 | CCTGAAGCTCCCTTGGTTC | AAATAGGACCCTCAAAAGAAATTG |
| [chemokine (C-X-C motif) ligand 2](http://www.ncbi.nlm.nih.gov/gene/20310) | Cxcl2 | CAGACTCCAGCCACACTTCA | GGTCTTCAGGCATTGACAGC |
| [Chemokine (C-X-C motif) ligand 3](http://www.ncbi.nlm.nih.gov/gene/330122) | Cxcl3 | AGGCTACAGGGGCTGTTGT | TGCCGCTCTTCAGTATCTTCT |
| [Interleukin 6](http://www.ncbi.nlm.nih.gov/gene/16193) | Il6 | AACGATGATGCACTTGCAGA | GGAAATTGGGGTAGGAAGGA |
| [Tumor necrosis factor](http://www.ncbi.nlm.nih.gov/gene/21926) | Tnf | CTCATGCACCACCATCAAGGACTC | TGGCTCTGTGAGGAAGGCTGTG |
| [Oncostatin M](http://www.ncbi.nlm.nih.gov/gene/18413) | Osm | TGCAGACACGGCTTCTAAGAAC | GAGTTGGAGCAGCCACGATT |
| [Interferon gamma](http://www.ncbi.nlm.nih.gov/gene/15978) | Ifng | GCGTCATTGAATCACACCTG | GAATCAGCAGCGACTCCTTT |
| [Interleukin 17A](http://www.ncbi.nlm.nih.gov/gene/16171) | Il17a | TCCAGAAGGCCCTCAGACTA | AGCATCTTCTCGACCCTGAA |
| [Interleukin 22](http://www.ncbi.nlm.nih.gov/gene/50929) | Il22 | GCTCAGCTCCTGTCACATCA | TTAGAAGGCAGGAAGGAGCA |
| [Intercellular adhesion molecule 1](http://www.ncbi.nlm.nih.gov/gene/15894) | Icam1 | TACATACGTGTGCCATGCCT | CCATCACGAGGCCCACAATG |
| [CD14 antigen](http://www.ncbi.nlm.nih.gov/gene/12475) | Cd14 | ACTGAAGCCTTTCTCGGAGC | TGAAAGCGCTGGACCAATCT |

**Table S2. List of the top-20 genes upregulated genes by dermal fibroblasts stimulated with a 10 % dilution of keratinocyte extract and 20 pg/ml of IL-1α.**

| **Gene Title** | **Gene Symbol** | **Affymetrix U219 array** | **Keratinocyte extract** | **IL-1α** |
| --- | --- | --- | --- | --- |
|  |  |  |  |  |
|  |  | (Probe Set ID) | (Fold change^1^) | (Fold change^1^) |
| Chemokine (C-X-C motif) ligand 8 | CXCL8 | 11718841_s_at | **162,68** | **181,94** |
| Chemokine (C-X-C motif) ligand 1 (melanoma growth stimulating activity, alpha) | CXCL1 | 11719366_s_at | **142,23** | **156,18** |
| Chemokine (C-X-C motif) ligand 2 | CXCL2 | 11744128_x_at | **85,16** | **99,30** |
| Chemokine (C-X-C motif) ligand 6 | CXCL6 | 11730801_at | **85,13** | **103,58** |
| Chemokine (C-X-C motif) ligand 3 | CXCL3 | 11728477_at | **70,19** | **83,18** |
| Vascular cell adhesion molecule 1 | VCAM1 | 11719675_a_at | **58,53** | **59,88** |
| Chemokine (C-X-C motif) ligand 10 | CXCL10 | 11720298_at | **56,06** | **121,82** |
| GTP cyclohydrolase 1 | GCH1 | 11721733_a_at | **55,85** | **67,16** |
| Interferon-induced protein with tetratricopeptide repeats 2 | IFIT2 | 11721873_at | **45,94** | **73,83** |
| Cytidine monophosphate (UMP-CMP) kinase 2, mitochondrial | CMPK2 | 11723105_at | **41,19** | **63,75** |
| GTP cyclohydrolase 1 | GCH1 | 11721734_s_at | **40,95** | **55,19** |
| Interferon induced, with helicase C domain 1 | IFIH1 | 11724346_a_at | **37,26** | **48,33** |
| Intercellular adhesion molecule 1 | ICAM1 | 11732999_a_at | **35,01** | **41,96** |
| Guanylate binding protein 1, interferon-inducible | GBP1 | 11752930_a_at | **34,04** | **42,90** |
| Chromosome 15 open reading frame 48 | C15orf48 | 11743350_a_at | **30,53** | **26,64** |
| 2'-5'-oligoadenylate synthetase 1 | OAS1 | 11719588_a_at | **27,10** | **47,59** |
| Interleukin 6 | IL6 | 11746463_a_at | **23,51** | **34,47** |
| Chemokine (C-C motif) ligand 20 | CCL20 | 11724828_at | **12,81** | **15,02** |
| Interleukin 1 beta | IL1B | 11719916_at | **9,24** | **17,74** |
| Chemokine (C-X-C motif) ligand 11 | CXCL11 | 11749245_a_at | **3,82** | **10,25** |

^1^ Data were extracted from the analysis of the transcriptional profiles of stimulated fibroblasts compared to unstimulated fibroblasts obtained by Affymetrix technology. Data are presented as gene expression fold change above unstimulated cells.

1. **Supplementary figures:**

**
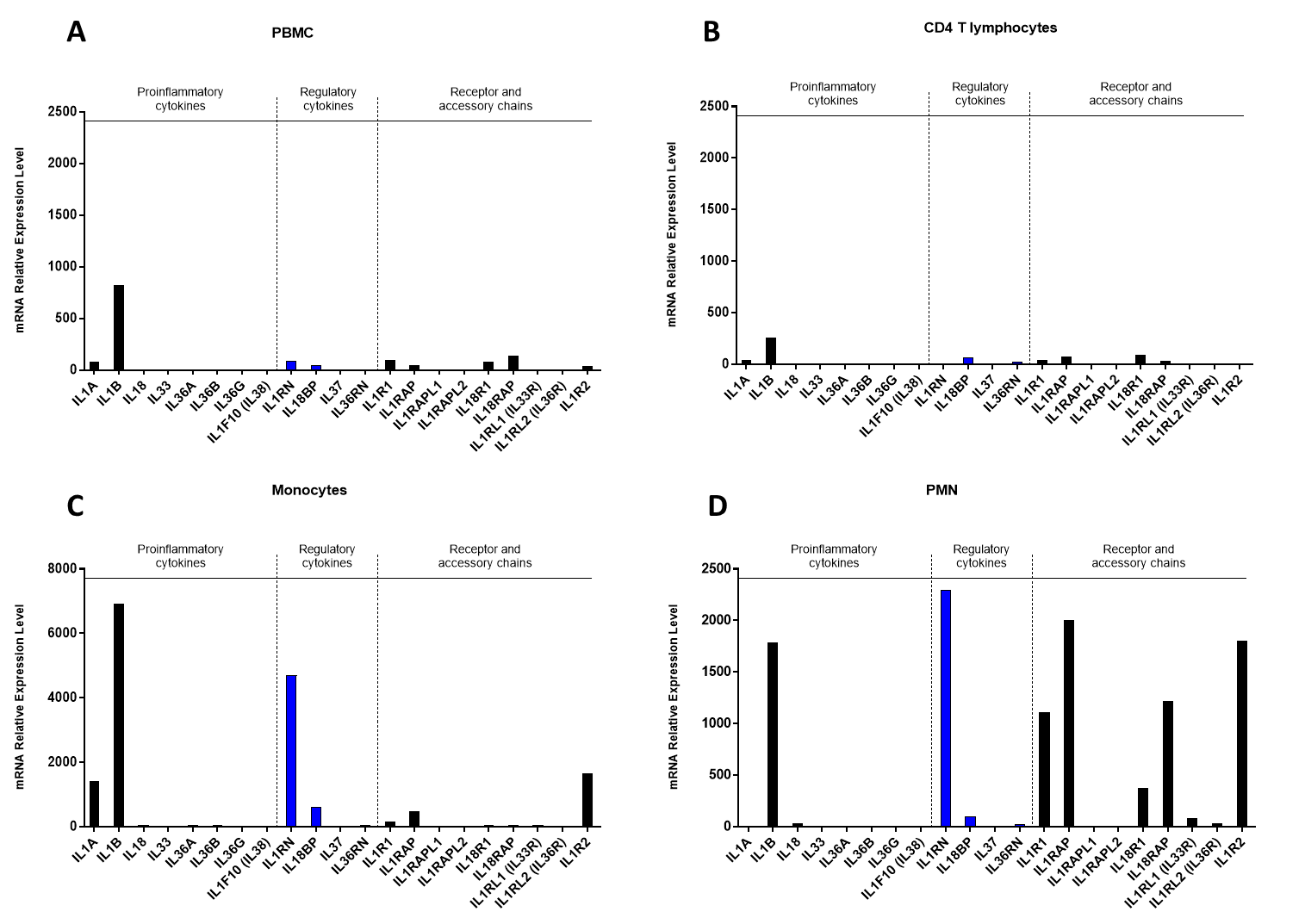
**

**Supplementary figure 1: Gene expression profiles of proinflammatory cytokines, regulatory cytokines and receptors of the IL-1 family by immune cells.** The expression of the IL-1 family members and their receptors was analyzed at the transcriptional level by Affymetrix analysis (hU219 array) after mRNA extraction of human peripheral blood mononuclear cells (A, PBMC), isolated CD4+ T cells (B) monocytes (C) and polymorphonuclear cells (D, PMN). Averaged data obtained from 2 independent cultures for each cell type are presented in relative expression of the gene of interest.

**
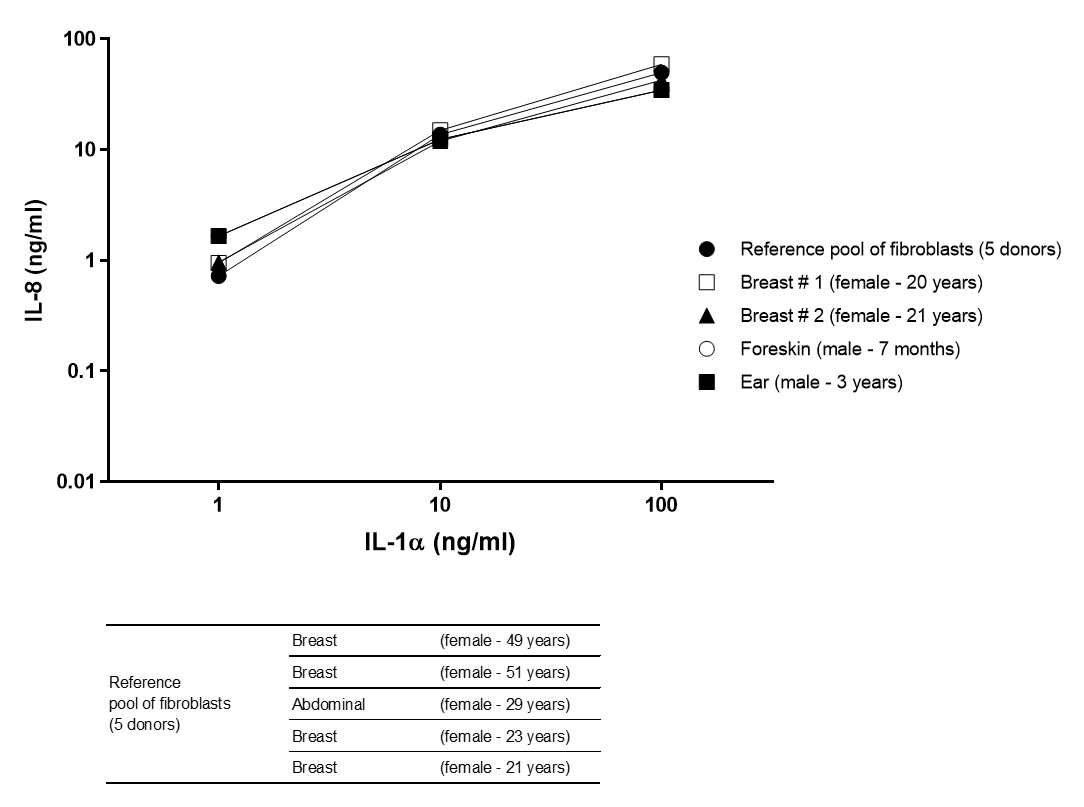
**

**Supplementary figure 2: Dermal fibroblasts isolated from different anatomic sites and different donors have similar capacity to respond to IL-1α.** For comparison, dermal fibroblasts were isolated from the skin of breast, foreskin, ear and abdomen of different donors with different ages and cultivated in the presence of increasing concentrations of IL-1α. The pool of fibroblasts isolated from five other donors, used in our *in vitro* studies, was also included as reference. The fibroblast response to IL-1α was evaluated by measuring IL-8 concentrations by ELISA in culture medium 24 h after cytokine stimulation.

**
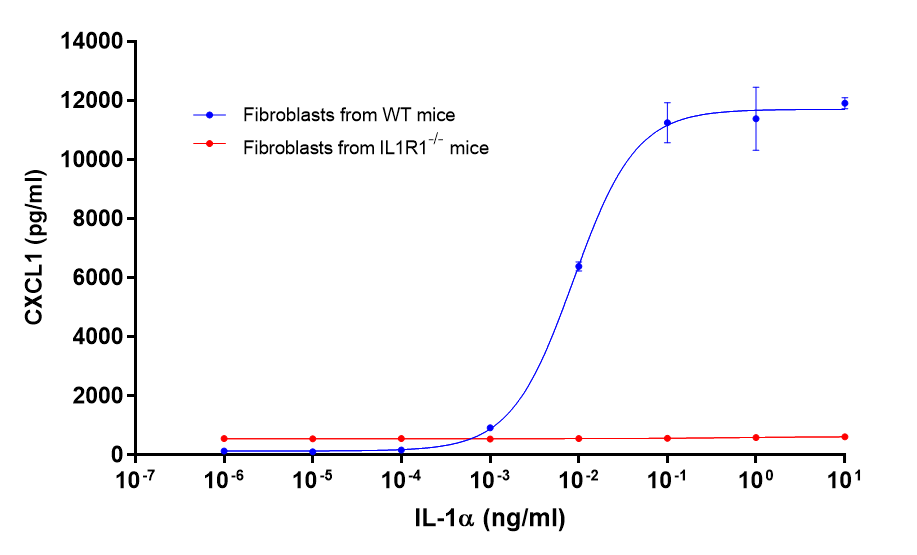
**

**Supplementary figure 3: Dermal fibroblasts from IL-1R1-deficient mice are unable to respond to IL-1α.** Dermal fibroblasts isolated from WT and IL-1R1-deficient mice (IL-1R1^-/-^) were stimulated with increasing concentrations of IL-1α for 24h before measuring CXCL-1 concentrations in culture medium by ELISA.
